# Wearable Sensor‐Based Prediction of Knee Joint Moments in Competitive Rugby Players During Multidirectional Movements

**DOI:** 10.1111/sms.70391

**Published:** 2026-10-30

**Authors:** Zixiang Gao, Robin Trama, David McNish, Michael Esposito, Jake Ruschkowski, Darren J. Stefanyshyn, John W. Wannop

**Affiliations:** ^1^ Human Performance Laboratory, Faculty of Kinesiology University of Calgary Calgary Canada; ^2^ Biomedical Engineering Graduate Program University of Calgary Calgary Canada

**Keywords:** explainable artificial intelligence, joint moments, machine learning, pedobarographic measurements, Rugby

## Abstract

This study aimed to develop an interpretable machine learning framework using plantar‐pressure sensors to estimate knee moments during rugby‐specific movements. A total of 31 North American competitive rugby players were recruited to perform four common rugby movements on an infilled artificial turf surface in a motion capture laboratory. Synchronized data acquisition was performed for plantar pressure, motion capture, and ground reaction forces. Knee moments were calculated via inverse dynamics. Genetic Algorithm‐Deep Forest Regression (GA‐DFR) was used to select plantar‐pressure sensors and predict resultant moments within participant‐level nested cross‐validation. Four alternative models predicted the resultant using the same layouts and participant splits. Complementary deep forest regression (DFR) models predicted the three moment components using these layouts. SHapley Additive exPlanations (SHAP) interpreted held‐out predictions. Layouts contained 7–18 sensors. Pooled resultant *R* ^2^ was 0.795, with root mean square error of 0.0512 N·m/N. DFR performed better than linear regression and the convolutional neural network, with no significant differences from the deep neural network or long short‐term memory network. Pooled *R* ^2^ was 0.806, 0.662, and 0.675 for flexion‐extension, adduction‐abduction, and internal‐external rotation moments, respectively. Resultant temporal SHAP contributions exceeded their stance‐wide mean during 9%–55% of stance, peaking at 24%. Reduced plantar‐pressure inputs supported estimation of resultant and direction‐specific knee‐moment waveforms in unseen rugby players.

## 1 Introduction

Rugby is a high‐intensity collision sport characterized by rapid accelerations, abrupt directional changes, and frequent physical impacts, placing mechanical demands on athletes' lower limbs. Epidemiological studies in English professional men's rugby union have identified knee injuries as a source of injury burden, with a match injury rate of 9.8 per 1000 player‐hours and a mean time loss of 50 days per injury [1]. In an earlier professional rugby cohort, anterior cruciate ligament (ACL) injuries accounted for 29% of days missed due to knee injuries [2].

In sports biomechanics, estimating knee joint moments during rugby‐specific tasks provides a measure of mechanical loading for movement assessment [3]. The three‐dimensional resultant joint moment represents the Euclidean magnitude of the sagittal‐, frontal‐, and transverse‐plane net joint moments [4]. It summarizes the overall net joint moment. The components describe the rotational demand in each direction. Traditionally, measuring these joint moments requires laboratory‐based motion capture and force plates to perform inverse dynamics calculations [4]. The reliance on laboratory instrumentation limits their use for field‐based load monitoring.

Wearable sensors combined with machine learning have provided an alternative for joint‐moment estimation in recent years. Stetter et al. estimated knee flexion and adduction moments from inertial sensors [3], while Mundt et al. and Dorschky et al. used neural networks to estimate lower‐limb kinetics from inertial measurements or simulated inertial signals [5, 6]. Plantar‐pressure‐based approaches have also been used to optimize sensor layouts for ankle‐moment prediction and to estimate knee moments during activities of daily living [7, 8]. Recurrent networks have further been applied to ankle‐biomechanics prediction [9]. Recent work has extended joint‐moment estimation to diverse cyclic and non‐cyclic activities. Adding simulated insole signals improved estimation relative to kinematic inputs alone in a multi‐activity study [10], and biological joint‐moment estimation has been incorporated into task‐agnostic exoskeleton control [11]. These studies differ in their target joints, movements, sensor inputs, and validation procedures. However, the feasibility and performance of these methods for the highly dynamic, multidirectional movements characteristic of rugby using a reduced pressure‐sensor set remain to be evaluated.

Therefore, the current study introduces an integrated framework combining genetic algorithm (GA)‐based sensor layout optimization with deep forest regression (DFR) modeling to predict knee resultant‐moment waveforms from plantar pressure data in rugby athletes. The resultant was the primary prediction target for sensor‐layout optimization and model comparison. Complementary analyses estimated the flexion‐extension, adduction‐abduction, and internal‐external rotation components using the resultant‐derived layouts to assess their direction‐specific performance. To identify the sensor locations and stance phases contributing to resultant predictions, SHapley Additive exPlanations (SHAP) was applied to the DFR model [12, 13]. The objectives of this research were twofold: (1) to establish a predictive and interpretable machine learning framework for estimating knee moments from pedobarographic measurements during rugby‐specific movements; and (2) to identify the pressure sensors and stance phases contributing most to resultant‐moment predictions of the data‐driven model.

## 2 Materials and Methods

### 2.1 Participants and Study Design

A total of 31 (age: 21.0 ± 2.3 years, height: 171.7 ± 7.3 cm, mass: 76.4 ± 13.4 kg, shoe size: 8.6 ± 1.5 US) competitive North American rugby players (9 male and 22 female) were recruited for this study. All players had more than 5 years of rugby experience and continued to train and compete; most were members of university rugby teams. All athletes reported no lower extremity or pelvic injuries within the past 6 months. Participants provided informed written consent before participating in the study. Ethical approval for this research was granted by the Conjoint Health Research Ethics Board at the University of Calgary (Ethics ID: REB22‐1046).

### 2.2 Experimental Protocol

To capture knee loading during sport‐specific maneuvers, we designed an experimental protocol comprising four fundamental rugby movements: a 90° cutting maneuver, a straight‐line sprint, a running pass with ball, and a 180° change‐of‐direction (COD) turn. These tasks were chosen to reflect common scenarios in match play that involve abrupt changes in momentum and actions known to elicit significant knee joint moments. The measurement section was covered with infilled artificial turf (FieldTurf, Tarkett Inc., Montreal, QC, Canada), with non‐infill turf in the surrounding areas to simulate a rugby training and match environment. The surface consisted of 60 mm RGF XM7 fibers installed over a 20 mm ProPlay shock pad. The infill system replicated realistic training and match environments, comprising a lower layer of sand (30 kg/m^2^, 20 mm) and an upper layer of styrene‐butadiene rubber (SBR) (11 kg/m^2^, 25 mm). All participants wore standardized boots (adidas), supplied in a complete size run to ensure proper fit. Reflective markers were affixed to the acromion processes, sternum, anterior and posterior superior iliac spines, iliac crests, and central sacrum, with bilateral placement at paired landmarks. Three additional markers were positioned on the posterior trunk near the spine. Additional markers were placed bilaterally at the medial and lateral femoral epicondyles and at the medial and lateral malleoli. Eight rigid marker clusters were attached bilaterally to the thighs, shanks, forefeet, and rearfoot. Each thigh, shank, and rearfoot cluster contained four markers, while each forefoot cluster contained three, giving 30 cluster markers in total. Participants were instructed to perform the tasks with maximal effort using their habitual technique. All analyses used the left foot.

An insole plantar pressure system containing 235 sensors (XSENSOR Technology Corporation, Canada, 150 Hz) was used to collect plantar pressure time‐series data. A 12‐camera high‐speed motion capture system (Vicon, Oxford, UK, 300 Hz) synchronized with a force plate (Kistler AG, Winterthur, Switzerland, 3000 Hz) was used to synchronously record marker trajectories and ground reaction force (GRF) signals. The acquisition procedure is illustrated in Figure 1A. The laboratory arrangement and marker configuration are shown in Figure S1 and Methods S1.

**FIGURE 1:**
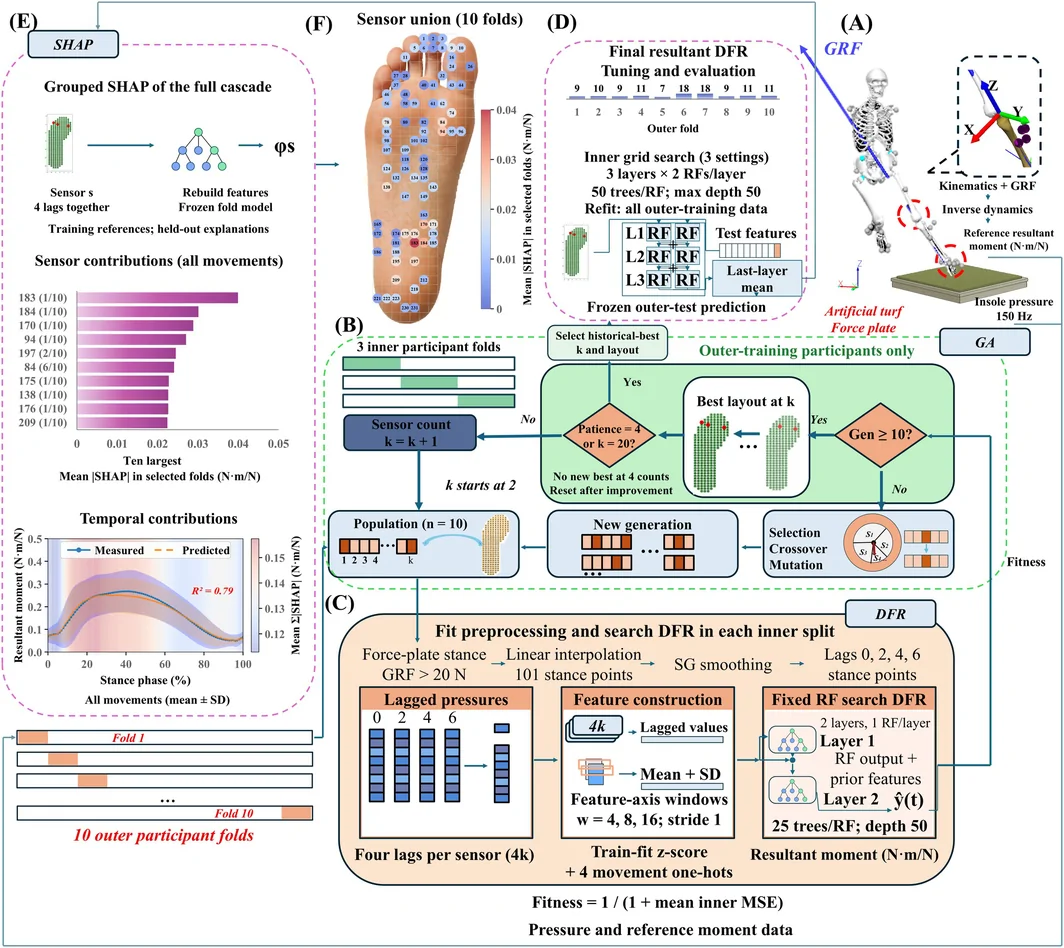
Acquisition, pressure‐feature construction, sensor selection, model tuning, and participant‐level evaluation. Pressure signals were stance‐normalized before smoothing and lag construction (Section 2.3; Methods S1–S3). (C) The fixed sensor‐search model, and (D) the final resultant configuration selected in all 10 folds: Three layers, two RFs per layer, 50 trees per RF, and maximum depth 50. Sensor selection, preprocessing, and tuning used outer‐training participants; outer‐test participants were held out for final evaluation. Component models used the same sensor layouts and were tuned separately. (E) Summarizes held‐out resultant‐model SHAP; parentheses denote selecting folds out of 10, and sensor means include only those folds. (F) The 80‐sensor union across the 10 layouts, each containing 7–18 sensors. DFR, deep forest regression; GA, genetic algorithm; RF, random forest.

### 2.3 Data Processing

#### 2.3.1 Preprocessing

Marker trajectories, GRF, and plantar pressure data were aligned using a shared trigger in Vicon Nexus (2.16.X64, Vicon Motion Systems Ltd., Oxford, UK). Marker trajectories and GRF signals were low‐pass filtered at 20 Hz using a bidirectional second‐order Butterworth filter. For each movement, stance onset and offset were defined by the force‐plate vertical‐force threshold of 20 N. The synchronized 150‐Hz pressure signals were segmented over this stance interval and linearly interpolated to 101 equally spaced stance points (0%–100%) for each trial. Inverse dynamics analysis was conducted using Visual3D (C‐Motion Inc., Germantown, MD, USA) to calculate the flexion‐extension (X), adduction‐abduction (Y), and internal‐external rotation (Z) net moments at the left knee. The knee resultant moment, calculated as the norm of the three‐dimensional (3D) knee moment vector [4], was normalized to body weight in newtons (N·m/N) and interpolated to 101 stance points in MATLAB (MathWorks, Natick, MA, USA). The interpolated resultant was smoothed using an 11‐point, second‐order Savitzky–Golay filter (Methods S1). Two experienced researchers supervised the collection of at least three trials per participant for each movement task. Trials were deemed invalid if visual inspection revealed (1) deliberate force‐plate targeting, (2) partial or edge contact of the stance foot with the platform perimeter, or (3) aberrant kinematics inconsistent with the participant's natural movement pattern. In total, 590 valid trials were analyzed (147 90° cutting, 145 sprinting, 148 running pass, 150 180° COD), yielding 59 590 frames of time‐series data.

#### 2.3.2 Model Inputs and DFR Construction

A DFR model learned the nonlinear mapping from plantar pressure features to knee‐moment time series (Figure 1). After interpolation to 101 points, pressure signals were smoothed independently within each trial using an 11‐point, second‐order Savitzky–Golay filter (derivative order 0; boundary mode interp). The current value for each selected sensor was concatenated with its values from the preceding 2, 4, and 6 stance‐normalized points. Because these offsets were applied after stance normalization, they correspond to 2, 4, and 6 percentage points of stance. Missing lagged values at the beginning of stance were padded with zeros; lags did not cross trial boundaries. Movement type was included as a one‐hot‐encoded input.

The DFR framework used a random‐forest cascade [7, 14]. For k selected sensors, the lagged‐pressure vector contained 4 k values, ordered by lag and sensor. At each prediction point, sliding‐window means and standard deviations were extracted across the lag‐sensor feature vector using widths of 4, 8, and 16 and a stride of one. The raw lagged values and window summaries were standardized using parameters estimated from the training data, then concatenated with the four movement indicators.

These features were used as inputs to the forest cascade. At each layer, in‐sample training predictions from the random forests were added as input features for the next layer. Held‐out data passed through the fitted layers without refitting. The final output was the average prediction from the forests in the last layer. Grid search compared (layers, forests per layer, trees per forest, maximum depth) settings of (2, 1, 25, 50), (3, 1, 50, 50), and (3, 2, 50, 50). Cascade depth was fixed within each candidate. The feature construction and cascade‐fitting procedure are detailed in Methods S2 and Table S1.

#### 2.3.3 Sensor Selection and Model Tuning

Model performance was evaluated using 10‐fold nested cross‐validation grouped by participant [15, 16, 17]. Within each outer‐training partition, three inner participant‐grouped folds were used for sensor‐count selection, sensor‐location optimization, learned preprocessing, and hyperparameter tuning. All data‐dependent procedures and model fitting were restricted to the outer‐training partition; the outer‐test partition was reserved for final evaluation.

To determine the optimal number of sensors, the algorithm initiated with a minimal configuration of two sensors and progressively increased the count (Figure 1). At each count, a GA optimized sensor locations using fitness‐proportionate roulette‐wheel selection [18]. The GA‐DFR sensor‐search framework followed Gong et al. [7]. Candidate layouts were evaluated using a fixed DFR configuration and the three inner validation folds. We converted the inner‐validation mean squared error (MSE) to a positive fitness for roulette‐wheel selection:

(1) $$\text{Fitness}\left(P\right)=\frac{1}{1+\mathrm{MSE}\left(P\right)}$$

Here, MSE(P) denotes the mean squared error between predicted and measured knee resultant moments, averaged across the three inner validation folds for layout P. No redundancy penalty was applied. This transformation preserves the ranking of layouts by MSE: lower MSE gives higher fitness.

The search stopped after four consecutive sensor counts without exceeding the historical best fitness. The layout with the highest inner fitness was selected for grid‐search hyperparameter tuning. Inner search and tuning evaluated every second stance frame. The chosen configuration and preprocessing were refitted using all outer‐training frames. The fitted model was then evaluated once on all 101 stance‐normalized frames of each trial from the previously unseen outer‐test participants. The search and tuning settings are detailed in Methods S3 and Table S1.

### 2.4 Data Analysis

#### 2.4.1 Predictive Performance and Model Comparison

To assess prediction accuracy, *R* ^2^, Pearson correlation coefficient (PCC), root mean square error (RMSE), and mean absolute error (MAE) were computed from held‐out predictions, both pooled and separately for each participant and trial. Pooled metrics were computed after concatenating all held‐out frames. Fold‐level metrics were computed separately in each outer test fold and summarized as mean and sample standard deviation (SD); participant‐ and trial‐level distributions were summarized as median and interquartile range (IQR). For the resultant, trial‐normalized RMSE, peak‐magnitude and peak‐timing errors, and participant‐level Bland–Altman agreement [19] were also evaluated (Methods S5). For each signed component, global maxima and minima were extracted independently from measured and predicted 101‐point waveforms. Absolute amplitude and timing errors compared corresponding extrema, using the first occurrence for exact ties (Methods S7).

The predictive performance of linear regression (LR), long short‐term memory network (LSTM), convolutional neural network (CNN), and deep neural network (DNN) was also evaluated. The models used the same outer participant splits and fold‐specific DFR‐selected sensor layouts, with preprocessing, tuning, and fitting performed within the training partitions (Methods S4 and Table S1). As a complementary analysis, separate DFR models predicted the three knee‐moment components. Each used the sensor layout selected for the resultant in its outer fold and was tuned and fitted for its own target (Methods S7).

For inference, each participant supplied one paired *R* ^2^, MAE, and RMSE value per model. Friedman tests compared the five models across the participants, with Holm correction across the three metrics. Follow‐up two‐sided Wilcoxon signed‐rank tests compared DFR with each alternative, with Holm correction across the 12 model‐metric comparisons. Kendall's W and rank‐biserial correlations were reported as effect sizes. Trials and frames were not treated as independent observations for statistical testing.

#### 2.4.2 SHAP Interpretation

SHAP was used as a post hoc interpretation method to characterize how the input features contributed to the model predictions [12, 13]. It was calculated for held‐out resultant predictions from the full fitted DFR cascades, grouping each physical sensor's four lagged inputs together and using reference data from outer‐training participants performing the same movement. Movement type was held fixed and was not attributed. The estimation procedure and equations are provided in Methods S6.

Spatial contributions to resultant predictions were summarized as mean absolute SHAP, averaged equally over folds selecting each sensor; selection frequency was calculated separately. Temporal contributions were calculated by summing absolute sensor attributions within each prediction and averaging at the same target stance frame. Lagged inputs were attributed jointly to that prediction frame. Both summaries were calculated across all movements and separately for each movement. For descriptive visualization, consecutive frames exceeding the temporal curve's mean across all 101 stance points were marked as above‐mean intervals (Table S10).

### 2.5 Declaration of the Use of Artificial Intelligence

ChatGPT‐5.5 (OpenAI, San Francisco, CA, USA) was used solely to assist with grammar and language refinement in parts of the manuscript and to refine auxiliary code for data preprocessing. All AI‐assisted codes were reviewed and verified by the authors. No artificial intelligence tool was used for the independent analysis or interpretation of data or the generation of scientific conclusions. The authors take full responsibility for the accuracy and integrity of the published work.

## 3 Results

### 3.1 Sensor Selection

Across the 10 outer folds, the selected layouts contained 7–18 sensors, with a median of 10.5. Eight folds met the stopping criterion of four consecutive counts without a new historical best. The remaining two folds reached the 20‐sensor search limit and each retained 18 sensors. In total, 80 distinct sensors were selected across the folds, with low pairwise overlap (mean Jaccard index 0.050). Sensor 84 was selected most frequently, appearing in six folds. Full layouts and test‐participant assignments are presented in Table S2.

### 3.2 Resultant‐Moment Prediction

Overall, DFR achieved a pooled *R* ^2^ of 0.795 and PCC of 0.892 across 59 590 held‐out frames, with RMSE of 0.0512 N·m/N and MAE of 0.0362 N·m/N. Participant‐level *R* ^2^ had a median of 0.800 (IQR 0.735–0.838), and trial‐level *R* ^2^ had a median of 0.784 (0.613–0.882).

As illustrated in Figure 2A1,A2, the model achieved an *R* ^2^ of 0.863 and a PCC of 0.930 for the 90° cutting movement, with RMSE of 0.0485 N·m/N and MAE of 0.0358 N·m/N. Cutting had the highest *R* ^2^ and PCC among the four movements.

**FIGURE 2:**
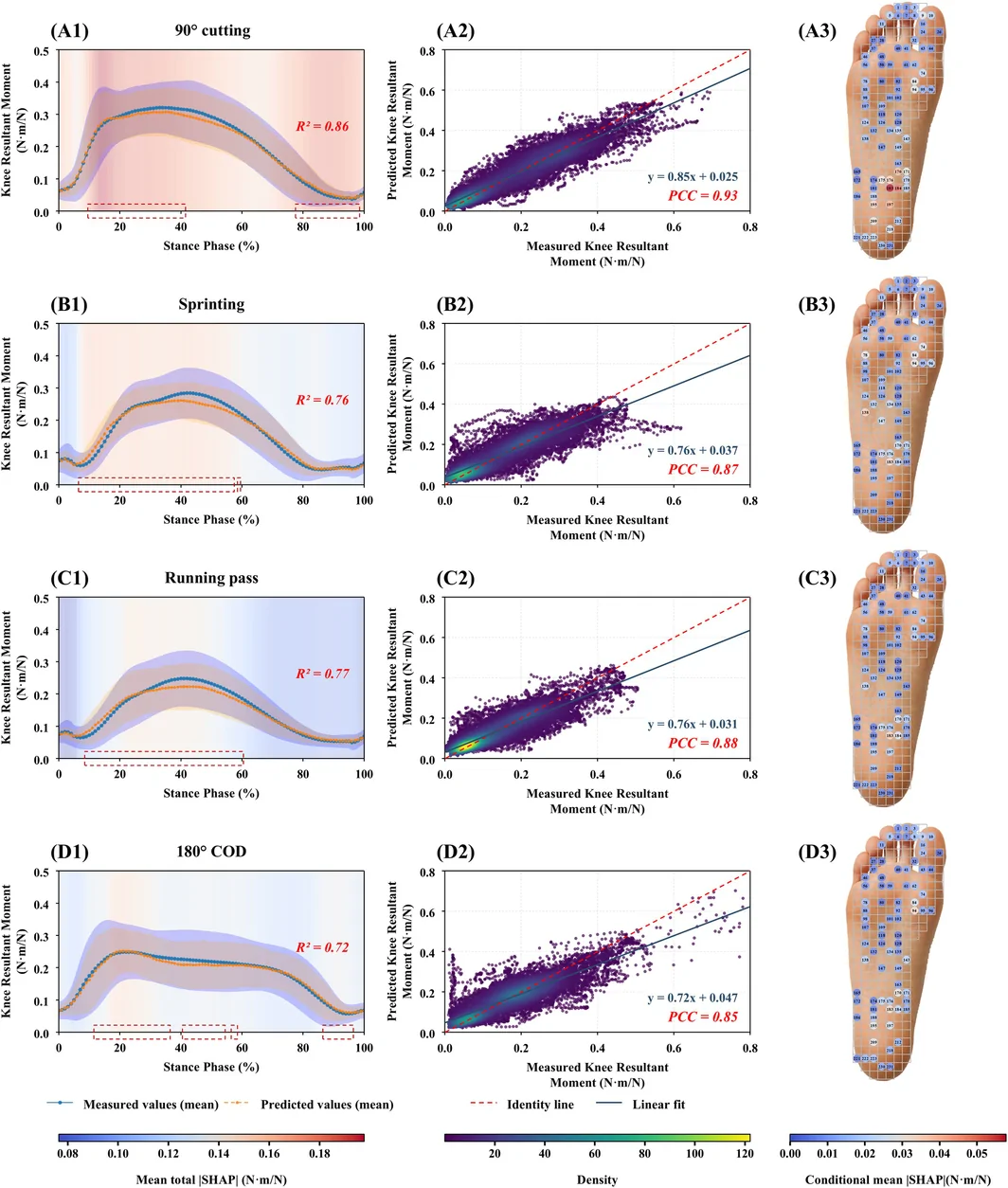
Held‐out knee resultant‐moment predictions and sensor contributions. Rows A–D show 90° cutting, sprinting, running pass, and 180° COD. (A1–D1) Measured (blue solid) and predicted (orange dashed) trial means with pointwise sample SD. Background color shows mean total absolute sensor SHAP; red dashed boxes mark above‐mean intervals. (A2–D2) Density scatter with identity and fitted regression lines. (A3–D3) Mean absolute sensor SHAP, averaged over folds selecting each sensor. Numbers are one‐based sensor IDs. *R* ^2^ and PCC use individual held‐out frames. Axes and the respective color scales are common across movements. Moments and SHAP are in N·m/N.

During sprinting (Figure 2B1,B2), the model achieved an *R* ^2^ of 0.759 and a PCC of 0.871, with RMSE of 0.0540 N·m/N and MAE of 0.0372 N·m/N.

As illustrated in Figure 2C1,C2, the model achieved an *R* ^2^ of 0.767 and a PCC of 0.877 for the running pass, with RMSE of 0.0463 N·m/N and MAE of 0.0329 N·m/N. This movement had the lowest RMSE and MAE among the four tasks.

As illustrated in Figure 2D1,D2, the model achieved an *R* ^2^ of 0.717 and a PCC of 0.847 for the 180° COD, with RMSE of 0.0555 N·m/N and MAE of 0.0390 N·m/N. This task had the lowest *R* ^2^ and the largest absolute errors.

For the resultant, trial‐level waveform‐normalized RMSE had a median of 15.2% (IQR 11.6%–19.9%). Median absolute peak‐magnitude error was 0.0329 (0.0145–0.0565) N·m/N, and median absolute peak‐timing error was 6.0 (2.0–13.8) percentage points of stance. Participant‐level peak agreement and full error distributions are reported in Tables S6 and S7 and Figures S2 and S3. After averaging repeated trials within each participant and movement, the mean predicted‐to‐measured between‐participant waveform SD ratios were 0.854 for cutting, 0.720 for sprinting, 0.821 for running pass, and 0.879 for 180° COD (Figure S4).

Overall and movement‐specific *R* ^2^, PCC, MAE, and RMSE values in the narrative and waveform figures use pooled held‐out frames for the relevant outcome and movement. Table 1 reports mean (sample SD) metrics calculated separately in the 10 outer test folds, with each fold weighted equally. Component metrics are pooled in Table 2, with fold summaries in Table S8. The four movements are compared together in Figure 2.

**TABLE 1:** Performance of knee resultant moment estimation across the pooled movements and four rugby‐specific movements using five data‐driven models.

Movement	Model	*R* ^2^	PCC	MAE	RMSE
Overall movements	DFR	0.795 (0.040)	0.893 (0.022)	0.036 (0.003)	0.051 (0.005)
	LR	0.598 (0.037)	0.775 (0.022)	0.054 (0.003)	0.072 (0.004)
	LSTM	0.803 (0.040)	0.899 (0.022)	0.037 (0.004)	0.050 (0.006)
	DNN	0.797 (0.043)	0.897 (0.022)	0.037 (0.003)	0.051 (0.006)
	CNN	0.730 (0.042)	0.859 (0.023)	0.043 (0.004)	0.058 (0.005)
90° cutting	DFR	0.859 (0.024)	0.931 (0.010)	0.036 (0.005)	0.049 (0.007)
	LR	0.679 (0.060)	0.833 (0.039)	0.057 (0.005)	0.073 (0.007)
	LSTM	0.858 (0.034)	0.929 (0.017)	0.037 (0.004)	0.049 (0.006)
	DNN	0.863 (0.022)	0.932 (0.012)	0.036 (0.003)	0.048 (0.004)
	CNN	0.802 (0.046)	0.905 (0.018)	0.043 (0.006)	0.058 (0.007)
Sprinting	DFR	0.760 (0.089)	0.877 (0.046)	0.038 (0.011)	0.054 (0.015)
	LR	0.553 (0.080)	0.753 (0.053)	0.057 (0.011)	0.074 (0.014)
	LSTM	0.800 (0.070)	0.904 (0.040)	0.037 (0.009)	0.049 (0.014)
	DNN	0.782 (0.079)	0.889 (0.042)	0.038 (0.010)	0.051 (0.014)
	CNN	0.705 (0.118)	0.857 (0.051)	0.044 (0.012)	0.059 (0.017)
Running pass	DFR	0.767 (0.066)	0.878 (0.036)	0.032 (0.004)	0.045 (0.006)
	LR	0.523 (0.087)	0.734 (0.054)	0.049 (0.004)	0.066 (0.007)
	LSTM	0.767 (0.120)	0.886 (0.054)	0.033 (0.006)	0.045 (0.008)
	DNN	0.764 (0.080)	0.881 (0.039)	0.034 (0.004)	0.045 (0.006)
	CNN	0.690 (0.083)	0.842 (0.043)	0.040 (0.004)	0.052 (0.005)
180° COD	DFR	0.706 (0.094)	0.846 (0.055)	0.039 (0.006)	0.054 (0.011)
	LR	0.491 (0.126)	0.728 (0.063)	0.054 (0.007)	0.071 (0.010)
	LSTM	0.701 (0.082)	0.850 (0.041)	0.041 (0.005)	0.055 (0.011)
	DNN	0.695 (0.147)	0.847 (0.075)	0.040 (0.008)	0.054 (0.014)
	CNN	0.617 (0.124)	0.808 (0.066)	0.047 (0.006)	0.062 (0.011)

*Note:* Values are means (sample SD) of metrics calculated separately in the 10 outer test folds.

**TABLE 2:** Pooled held‐out performance for direction‐specific knee moments.

Movement	Component	*R* ^2^	PCC	MAE	RMSE
90° cutting	Flexion–extension	0.859	0.927	0.0396	0.0540
	Adduction–abduction	0.645	0.804	0.0314	0.0444
	Internal–external rotation	0.711	0.844	0.0174	0.0293
Sprinting	Flexion–extension	0.771	0.878	0.0440	0.0653
	Adduction–abduction	0.524	0.724	0.0235	0.0356
	Internal–external rotation	0.579	0.763	0.0116	0.0173
Running pass	Flexion–extension	0.807	0.899	0.0371	0.0519
	Adduction–abduction	0.462	0.682	0.0239	0.0337
	Internal–external rotation	0.644	0.802	0.0145	0.0210
180° COD	Flexion–extension	0.742	0.862	0.0412	0.0584
	Adduction–abduction	0.652	0.808	0.0328	0.0455
	Internal–external rotation	0.649	0.810	0.0207	0.0329

*Note:* Each metric is calculated after pooling all held‐out frames for the indicated movement and component. RMSE and MAE are in N·m/N. Component models used the sensor layout selected for the resultant in each fold.

### 3.3 Comparison With Alternative Models

Table 1 presents the performance of knee resultant moment estimation across the pooled movements and four rugby‐specific movements (90° cut, sprinting, running pass, and 180° COD) using five data‐driven models. Pooled *R* ^2^ values for DFR, LR, LSTM, DNN, and CNN were 0.795, 0.599, 0.801, 0.797, and 0.731, respectively (Table S3). LSTM and DNN achieved slightly higher pooled *R* ^2^ values, whereas DFR had a slightly lower pooled MAE.

Participant‐paired Friedman tests indicated differences among models for *R* ^2^ and RMSE (χ^2^(4) = 88.748, Kendall's *W* = 0.716) and for MAE (χ^2^(4) = 95.252, *W* = 0.768); all Holm‐adjusted *p* values were < 0.001. In the subsequent paired comparisons, DFR had better *R* ^2^, RMSE, and MAE than LR and CNN (all adjusted *p* < 0.001), but differences from DNN and LSTM were not statistically significant (all adjusted *p* = 1.000). Full test statistics and rank‐biserial effect sizes are provided in Tables S4 and S5.

### 3.4 Complementary Direction‐Specific Prediction

DFR achieved pooled *R* ^2^ values of 0.806, 0.662, and 0.675 for flexion‐extension, adduction‐abduction, and internal‐external rotation moments, respectively. The corresponding PCC values were 0.898, 0.814, and 0.822, and RMSE values were 0.0576, 0.0402, and 0.0259 N·m/N. Measured and predicted component waveforms for the four movements are shown in Figure 3.

**FIGURE 3:**
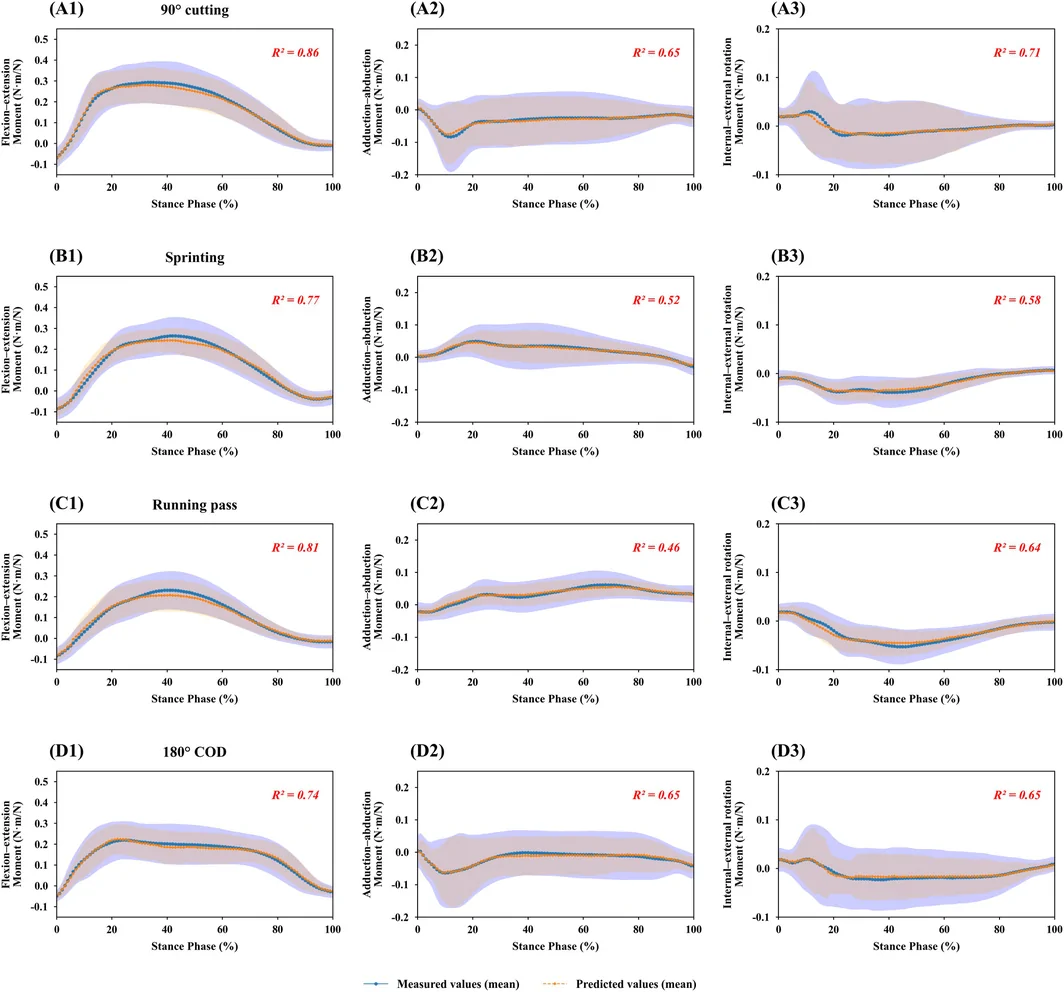
Held‐out predictions of the three knee‐moment components across the four rugby movements. Rows A–D show 90° cutting, sprinting, running pass, and 180° COD. Columns 1–3 show flexion‐extension, adduction‐abduction, and internal‐external rotation moments, respectively. Blue solid and orange dashed curves show measured and predicted trial means; shading shows pointwise sample SD. *R* ^2^ uses all individual held‐out frames. Each component model used the sensor layout selected for the resultant in its fold and was tuned separately using only training participants. Each component column uses the same y‐axis limits. Moments are in N·m/N.

Movement‐specific *R* ^2^ ranged from 0.742 to 0.859 for flexion‐extension, 0.462 to 0.652 for adduction‐abduction, and 0.579 to 0.711 for internal‐external rotation (Table 2). Flexion‐extension moments had the highest *R* ^2^ in each movement. Cutting had the highest *R* ^2^ for flexion‐extension and internal‐external rotation, while the 180° COD had the highest *R* ^2^ for adduction‐abduction. Adduction‐abduction *R* ^2^ was lowest during the running pass, and internal‐external rotation *R* ^2^ was lowest during sprinting.

Table 3 summarizes absolute amplitude and timing errors for the component maxima and minima across all 590 held‐out trials. Timing errors were larger for adduction‐abduction maxima and internal‐external rotation minima than for their opposite extrema. Table S9 reports trial‐level distributions overall and by movement, and Figure S5 shows participant‐level median errors. The measured flexion‐extension minimum occurred at a stance endpoint in 79.2% of trials.

**TABLE 3:** Amplitude and timing errors of knee‐moment component extrema.

Component	Extremum	Absolute amplitude error (N·m/N)	Absolute timing error (stance pp)
Flexion‐extension	Maximum	0.0311 [0.0142, 0.0549]	6.0 [2.0, 14.8]
Flexion‐extension	Minimum	0.0218 [0.0092, 0.0390]	1.0 [0.0, 3.0]
Adduction‐abduction	Maximum	0.0195 [0.0084, 0.0373]	7.5 [2.0, 28.0]
Adduction‐abduction	Minimum	0.0190 [0.0087, 0.0358]	3.0 [1.0, 16.0]
Internal‐external rotation	Maximum	0.0092 [0.0040, 0.0195]	3.0 [1.0, 13.0]
Internal‐external rotation	Minimum	0.0134 [0.0053, 0.0239]	11.0 [4.0, 21.0]

*Note:* Values are median [Q1, Q3] across all 590 held‐out trials from 31 participants. Maxima and minima were identified independently in measured and predicted signed waveforms over all 101 stance points; exact ties use the first occurrence. Errors are absolute differences in extremum amplitude and location. Timing errors are expressed in percentage points of stance. Extrema include the stance endpoints (0% and 100%).

### 3.5 Spatial and Temporal Contributions to Resultant Predictions

In the pooled SHAP summary, sensor 183 had the largest conditional mean absolute contribution (Figure 1E; Figure S6B). Temporal contributions exceeded their stance‐wide mean from 9% to 55% of stance, peaking at 24% (Figure S6A and Table S10).

Movement‐specific spatial contributions are compared using a common color scale in Figure 2A3–D3. Sensor 183 had the largest conditional mean absolute SHAP value during cutting, running pass, and the 180° COD; sensor 138 had the largest value during sprinting. Both sensors were selected in one fold, whereas sensor 84 was selected in six folds. Movement‐specific temporal intervals and their maxima are reported in Table S10.

## 4 Discussion

This study developed an explainable machine learning approach to predict knee resultant‐moment waveforms in competitive rugby athletes performing sport‐specific movements using reduced plantar pressure data. With 7–18 sensors selected within each training partition, DFR achieved a pooled *R* ^2^ of 0.795 in previously unseen players. DFR performed better than LR and CNN on the shared layouts, while differences from DNN and LSTM were not statistically significant. Complementary component predictions had the highest *R* ^2^ for flexion‐extension. Pressure information from early‐to‐middle stance contributed most to resultant predictions overall, with later stance also contributing during changes of direction.

The advantage of DFR over LR suggests that a nonlinear mapping was useful for relating pressure inputs to knee moments. Neural‐network studies of lower‐limb kinetics also support this modeling approach [5, 6]. The cascade structure of DFR can handle complex, non‐linear relationships [14], while DNN and LSTM provide alternative nonlinear mappings for the same inputs. LSTM achieved a higher prediction performance during sprinting, whereas DFR had a slightly higher performance during turning. These differences were small compared with their gaps from LR.

The component analysis adds direction‐specific information to the resultant‐moment estimates (Figure 3). The components show the sagittal‐, frontal‐, and transverse‐plane rotational demands that contribute to the resultant. Erhart‐Hledik et al. reported changes in the relative contributions of knee flexion and adduction moments during walking after ACL reconstruction, with no significant change in the first total‐moment peak [20]. The lower *R* ^2^ values for adduction‐abduction and internal‐external rotation show that prediction accuracy varies with moment direction; these components also had smaller absolute errors and waveform magnitudes. The extrema analysis assessed recovery of individual maxima and minima alongside waveform agreement. Most flexion‐extension minima occurred at the start or end of stance, so their small timing errors mainly reflect agreement in endpoint timing.

Within the present modeling framework, inner‐validation fitness did not consistently improve when additional sensors were introduced. Similarly, Gong et al. obtained their highest optimization fitness with nine pressure sensors when estimating ankle moments across three walking speeds [7]. Both studies support optimizing sensor number and location for the target movements. The preferred locations changed when the training players changed, with little overlap between layouts. Eils et al. reported distinct regional pressure patterns during running, cutting, sprinting, and kicking in soccer players [21]. Differences among players in these task‐dependent patterns may contribute to variability in the selected layouts.

Across the pooled analysis and three of the four movement tasks, sensor 183 in the heel region had the highest conditional mean absolute SHAP value. In contrast, task‐specific variations were evident among other sensors. During sprinting, sensor 138 had the highest contribution. Both sensors were selected in one fold, whereas sensor 84 in the medial forefoot was selected in six folds. Ogasawara et al. showed that the GRF acting point was associated with combined knee valgus and tibial internal‐rotation moments during early cutting [22]. The high conditional contribution of heel sensor 183 may reflect information about where load is applied under the foot.

Temporal decomposition of the stance phase revealed higher contributions during early‐to‐middle stance in the pooled analysis. This pattern was most apparent during sprinting and running pass, whereas cutting and turning also drew useful information from later stance. The temporal pattern reflects contributions from both current and lagged pressure values to predictions at each stance point.

### 4.1 Practical Applicability

The current framework estimates resultant‐moment waveforms from reduced pressure measurements for completed cutting, sprinting, passing, and turning trials in unseen players. These estimates describe the magnitude and timing of net rotational demand during each movement. The component estimates describe direction‐specific moments, with prediction accuracy reported separately. For instrumented‐insole development, the fold‐specific layouts and SHAP maps identify candidate sensing regions. Selection frequency shows how often a sensor was selected across folds. Conditional SHAP indicates its contribution in the folds where it was included.

This study has several limitations. (1) Net knee moments summarize rotational demands across the joint; individual tissue and contact forces depend on muscle and other anatomical contributions [23, 24]. (2) Predicted between‐participant resultant‐waveform variability was lower than measured variability, especially during sprinting, limiting sensitivity to individual loading characteristics. (3) Data from male and female rugby players were included to train the model, and previous ACL injury history was not recorded. The model comparison and component analyses used DFR‐selected resultant layouts, so their performance reflects this shared‐layout strategy. Stance normalization and within‐trial filtering used complete trials, so the present evaluation was offline.

## 5 Conclusion

This study developed an interpretable machine learning framework to estimate knee resultant‐moment waveforms in competitive rugby players from reduced plantar pressure data and movement type. Participant‐level nested cross‐validation supported prediction in previously unseen players using fold‐specific layouts of 7–18 sensors. Complementary analyses extended the estimates to the three moment components, with the highest *R* ^2^ for flexion‐extension. SHAP identified sensor contributions conditional on selection and greater overall temporal contributions during early‐to‐middle stance.

## 6 Perspective

Future studies should test a fixed sensor layout in independent players, footwear, and playing conditions, with calibration and sensitivity to changes in knee moments assessed within individual players. Sensor layouts optimized separately for the component moments could establish whether the frontal‐ and transverse‐plane predictions benefit from different pressure measurements. Combining pressure with kinematic or electromyographic (EMG) inputs should be evaluated for directional prediction during multidirectional tasks [10]. Reduced‐insole prototypes should evaluate heel and medial‐forefoot coverage while preserving pressure information throughout contact. Real‐time applications require causal signal processing and event detection that do not rely on a completed stance interval. Validation in sex‐specific cohorts and players with a defined injury or surgical history would extend the target population. Combining moment estimation with musculoskeletal modeling could examine muscle contributions to tibiofemoral loading [23, 24].

## Funding

The authors have nothing to report.

## Conflicts of Interest

The authors declare no conflicts of interest.

## Supporting information


**Figure S1:** Laboratory arrangement and marker configuration.
**Figure S2:** Trial‐level waveform and peak‐error distributions for the four rugby‐specific movements.
**Figure S3:** Participant‐level Bland–Altman agreement for peak knee resultant moment by movement.
**Figure S4:** Between‐participant variability in measured and predicted knee resultant‐moment waveforms.
**Figure S5:** Participant‐level errors in signed knee‐moment component extrema.
**Figure S6:** Pooled temporal and spatial contributions to held‐out resultant‐moment predictions.
**Table S1:** Sensor‐selection and model‐training settings.
**Table S2:** Outer test participants and selected sensor layouts.
**Table S3:** Pooled held‐out model performance.
**Table S4:** Paired participant‐level omnibus model comparisons.
**Table S5:** Paired comparisons of DFR with alternative models.
**Table S6:** Participant and trial distributions of DFR prediction error.
**Table S7:** Waveform normalization and peak agreement.
**Table S8:** Fold‐level prediction accuracy for the three knee‐moment components.
**Table S9:** Amplitude and timing errors of knee‐moment component extrema.
**Table S10:** Stance intervals with above‐mean sensor contribution to resultant predictions.

## Data Availability

The data that support the findings of this study are available from the corresponding author upon reasonable request.
