# Supplementary material for: Wearable Sensor‐Based Prediction of Knee Joint Moments in Competitive Rugby Players During Multidirectional Movements
Source: Scand J Med Sci Sports. 2026 Oct 30;36(11):e70391. doi: 10.1111/sms.70391 (PMC13721775; doi:10.1111/sms.70391)
Supplement: Supplementary file 1 [file SMS-36-e70391-s001.docx]

**Supplementary Material**

**Wearable sensor-based prediction of knee joint moments in competitive rugby players during multidirectional movements**

Knee moments and their absolute errors are normalized to body weight and reported in N·m/N.

## **Supplementary Methods**

## **S1 Experimental protocol and signal processing**

Participants performed the four tasks with maximal effort using their habitual technique. The 90° cutting and 180° change-of-direction (COD) labels denote nominal task directions, with self-selected foot-contact angles. The left foot was analyzed for all tasks, and the running pass was directed to the right.

The artificial-turf platform over the force plate had the same footprint as the plate and was fixed using large screws. An approximately 1 cm gap separated it from adjacent turf. The force plate was zeroed after installation of the platform and turf. Supplementary Figure S1 shows the laboratory arrangement, platform dimensions, and marker configuration.

Marker trajectories, ground reaction forces (GRF), and plantar pressure data were collected, with all data streams aligned based on a shared trigger in Vicon Nexus (2.16.X64, Vicon Motion Systems Ltd., Oxford, UK). Marker trajectories and ground-reaction-force signals were low-pass filtered at 20 Hz using a second-order Butterworth filter applied bidirectionally, resulting in an effective fourth-order zero-phase response. Stance onset and offset were defined by the synchronized force-plate vertical-force threshold of 20 N. The corresponding 150-Hz pressure interval was segmented and linearly interpolated to 101 equally spaced points spanning 0-100% of stance. Pressure smoothing and lag generation were performed after this interpolation. Inverse dynamics analysis was conducted using Visual3D (C-Motion, Inc., Germantown, MD, USA) to calculate joint moments at the left knee. The knee resultant moment was calculated as the norm of the three-dimensional (3D) knee moment vector, derived from its components in the sagittal, frontal, and transverse planes at each time point [1]. The resultant was then normalized by each participant's body weight expressed in newtons. The resulting unit was N·m/N, i.e., moment in N·m divided by body weight in N. The resultant time series was interpolated to 101 points over stance (MathWorks, Natick, MA, USA) and then smoothed using a Savitzky-Golay filter with an 11-point window, polynomial order 2, derivative order 0, and boundary mode ‘interp’ (SciPy).

## **S2 Pressure inputs and deep forest regression (DFR) construction**

After linear interpolation to 101 stance points, pressure signals were filtered independently within each trial using an 11-point, second-order Savitzky-Golay filter (derivative order 0; boundary mode interp). The current pressure and values from the preceding 2, 4, and 6 stance-normalized points were concatenated for each selected sensor, with zero padding at stance start and no crossing of trial boundaries. These lags correspond to 2, 4, and 6 percentage points of stance; for a trial of stance duration T, they span 0.02T, 0.04T, and 0.06T seconds. For a layout of k sensors, the pressure vector contained 4k values, ordered first by lag (0, 2, 4, 6) and then by ascending sensor index. Sliding-window means and standard deviations (SD) were calculated across this feature vector at each prediction point using widths of 4, 8, and 16 and a stride of one. Windows wider than 4k were omitted. Window SD used ddof = 0. These summaries formed the engineered multi-scale features. Pressure-derived features were standardized using parameters fitted to the training participants, and movement type was encoded using a one-hot encoder fitted to the training participants.

At each cascade layer, one or two random forests generated predictions that were appended to the feature matrix for the following layer. During fitting, the appended values were the layer's in-sample training predictions. During evaluation, the fitted layers were applied sequentially without refitting. The final output was the average prediction from the forests in the last layer. The complete feature-extraction and cascade-fitting procedure was repeated within the relevant training partition. Inner-validation and outer-test participants were processed using the corresponding fitted pipeline. Forests were scikit-learn RandomForestRegressor models, with random_state = 42 + 100 × layer + forest, where layer and forest indices start at zero. The number of cascade layers was fixed for each tuning candidate. All 10 final resultant models used three layers, two forests per layer, 50 trees per forest, and maximum depth 50. The component models selected their own configurations within the same candidate grid.

## **S3 Sensor selection and model training**

The 31 participants were grouped by identifier. Each participant was tested in exactly one of 10 outer folds; all 590 trials were retained. Three inner participant-grouped folds were used within each outer-training set. Data-dependent scaling and movement encoding were fitted on the corresponding training partition. Pressure filtering and lag construction were applied independently within each trial.

The sensor search started at 2 sensors. At each count, a genetic algorithm (GA) searched sensor locations using a population of 10 candidates for 10 generations, with one elite candidate retained. Roulette-wheel selection assigned each candidate a probability proportional to its fitness. Crossover probability decreased from 0.6 to 0.3, while mutation probability increased from 0.01 to 0.1. The search model used two cascade layers, one forest per layer, 25 trees per forest, and a maximum tree depth of 50. Fitness was 1/[1 + MSE(P)], with knee resultant-moment mean squared error (MSE) averaged across the three inner validation folds. The correlation-based redundancy coefficient was zero.

After each sensor count, its best fitness was compared with the best fitness at all previously evaluated counts. A strict improvement reset the no-improvement counter to zero; equality or a lower value increased the counter by one. The no-improvement stopping criterion was four consecutive counts without a new historical best. The sensor layout associated with the highest inner fitness score was selected for subsequent model tuning and fitting. On that layout, grid search compared (layers, forests per layer, trees per forest, maximum depth) settings of (2, 1, 25, 50), (3, 1, 50, 50), and (3, 2, 50, 50). The chosen configuration and preprocessing were refitted using all outer-training frames. The fitted model was then evaluated once on all 101 stance-normalized frames of each trial from the outer-test participants. The base random seed was 42.

## **S4 Alternative prediction models**

All comparison models received the same fold-specific DFR-selected physical sensors, engineered lag/window features, and movement one-hot input. Linear regression (LR) and deep neural network (DNN) operated on frame-wise features. Long short-term memory network (LSTM) and convolutional neural network (CNN) operated on the sequence of these features within each 101-point trial. The LSTM was unidirectional, and both CNN layers used left padding only. Prediction was evaluated offline because upstream stance normalization and within-trial filtering used complete trials.

Neural networks used Adam, training-target centering/scaling, and MSE loss. The target mean and SD were fitted using training values and predictions were returned to the target scale. Inner validation selected the configuration and stopping epochs; final training used the median inner-fold best epoch (rounded) and all outer-training frames. Search and inner scoring used a frame stride of two, with early stopping based on inner-validation participants.

## **S5 Prediction accuracy and statistical analysis**

To assess prediction accuracy, the coefficient of determination (R²), Pearson correlation coefficient (PCC), root mean square error (RMSE) and mean absolute error (MAE) were computed from the held-out predictions. Pooled metrics were computed once from all concatenated outer-test frames; participant metrics combined the held-out frames for that participant; trial metrics used each 101-point waveform separately. Fold-level metrics were computed separately for the 10 outer test sets and summarized as their mean and sample standard deviation (ddof = 1). Fold summaries weight folds equally; pooled MAE weights folds by their frame counts, pooled RMSE takes the square root after pooling squared errors, and pooled R² uses variation about the pooled outcome mean; participant- and trial-level distributions were summarized as median and interquartile range (IQR). Trial waveform-normalized root mean square error (NRMSE) was 100 × RMSE divided by that trial's observed peak-to-peak range. For the resultant, peak-magnitude error compared the maxima of predicted and measured waveforms, and peak-timing error compared their locations in percentage points of stance. Bland-Altman analysis used one mean trial peak per participant and movement; limits of agreement were the mean difference ± 1.96 times the sample standard deviation of the differences [2].

Participant-paired statistical tests are described in main Section 2.4.1. The fitted models shared overlapping outer-training sets.

## **S6 Spatial and temporal model interpretation**

For the resultant, SHapley Additive exPlanations (SHAP) quantified sensor contributions to predictions from the full fitted DFR cascade [3]. Grouped antithetic permutation Shapley estimation used 64 permutations per outer-test prediction and 32 fixed outer-training reference rows per movement. Reference sampling balanced participants and stratified stance phase. All four lagged values of a physical sensor formed one group. Masking replaced that group with values from a same-movement reference; the engineered window features were then recomputed and passed through the fitted scaler and complete cascade. Movement type remained fixed, so the attributions describe sensor contributions conditional on movement.

The Shapley value for physical-sensor group i was defined as follows [3]:

$\phi_{i}=\sum_{S\subseteq F\setminus\{i\}} \frac{|S|!(|F|-|S|-1)!}{|F|!}[v(S\cup\{i\})-v(S)]$	(S1)

F denotes the selected sensor groups, and S is a subset excluding i. The value v(S) is the mean full-cascade output over the specified training references when groups in S retain their observed values and the remaining groups take reference values; movement is unchanged. A signed Shapley value describes a change in the prediction relative to this reference. To quantify the mean absolute contribution of sensor i within fold f and movement a, its absolute Shapley values were aggregated as:

$A(i,f,a) = \frac{\Sigmaₙ |\varphi(i,n,f,a)|}{N(f,a)}$	(S2)

N(f,a) is the number of held-out predictions for fold f and movement a. Spatial mean absolute SHAP values are averaged equally across folds selecting each sensor. For Figure 1E-F and Supplementary Figure S6B, a mean is calculated for each fold across all held-out frames and movements, then averaged across the folds in which the sensor was selected. Figure 1E gives selecting-fold counts in parentheses. Temporal curves sum absolute sensor attributions within each prediction and average them at the target stance point, across all 590 trials for Supplementary Figure S6A or within each movement for Figure 2A1-D1. All four lags contribute jointly to that target point. Red dashed boxes mark consecutive points above the curve's arithmetic mean over 101 stance points. This descriptive threshold is applied separately to the pooled curve and each movement, retaining gaps and isolated points (Supplementary Table S10). Figure 2A3-D3 compares the four movement-specific sensor maps using a common color scale.

The random-number seed was fixed at 20260905 for the primary permutation estimates and 20260906 for a repeat using the same references and 101 equally spaced ordered outer-test rows per movement and fold. Sensor-rank correlation and relative L1 difference in mean absolute attribution quantified sensitivity to the permutation seed.

## **S7 Knee-moment component analysis**

In the complementary component analysis, separate DFR models predicted knee flexion-extension, adduction-abduction, and internal-external rotation moments. Each component model used the sensor layout selected for the resultant in its outer fold and was tuned for its own target using the same inner participant splits.

Global maxima and minima were extracted independently from measured and predicted held-out waveforms over all 101 stance points. Absolute amplitude and timing errors compared corresponding extrema values and locations, with timing expressed in percentage points of stance and the first occurrence used for exact ties. Supplementary Figure S5 summarizes each participant's median error across their available trials. Main Table 3 summarizes amplitude and timing errors across all 590 held-out trials. Supplementary Table S9 reports these results overall and by movement and includes the fraction of measured extrema at stance endpoints (0% or 100%).

## **Computational implementation**

The models were run on the University Advanced Research Computing (ARC) cluster with 12 central processing unit (CPU) workers per outer-fold job.

## **References for supplementary methods**

1. D. A. Winter, *Biomechanics and Motor Control of Human Movement*, 4th ed. (John Wiley & Sons, 2009).

2. J. M. Bland and D. G. Altman, “Statistical Methods for Assessing Agreement Between Two Methods of Clinical Measurement,” *The Lancet* 1, no. 8476 (1986): 307–310.

3. S. M. Lundberg and S.-I. Lee, “A Unified Approach to Interpreting Model Predictions,” in *Advances in Neural Information Processing Systems 30* (Curran Associates, 2017), 4765–4774.

## **Supplementary Results**

## **Knee-moment component prediction**

The pooled knee-moment component results and measured/predicted waveforms are presented in main Section 3.4, Table 2, and Figure 3. Supplementary Table S8 reports the mean (sample SD) across the 10 outer folds separately from those pooled values.

Main Table 3 summarizes trial-level extrema errors across all movements; Supplementary Table S9 reports the overall and movement-specific distributions, and Supplementary Figure S5 compares participant-level median errors.

## **Stability of the SHAP estimates**

For resultant SHAP, sensor-rank correlations across 40 fold-movement checks ranged from 0.955 to 1.000; relative L1 differences in mean absolute attribution ranged from 0.007 to 0.036.

## **Supplementary Table S1. Sensor-selection and model-training settings**

| **Item** | **Setting used** |
| --- | --- |
| Outer / inner validation | 10 participant GroupKFold splits / 3 inner participant GroupKFold splits; complete trials remain together. |
| Inputs | 235 candidate pressure channels; trial-wise Savitzky-Golay filtering; lagged-pressure and multi-scale features; training-fitted standardization and movement encoding (Section S2). |
| GA | Counts 2-20; population 10; 10 generations; elite 1; crossover 0.6→0.3; mutation 0.01→0.1; fixed seed 42. Fitness = 1/(1 + mean inner MSE); redundancy coefficient 0. |
| Count stopping | No-improvement criterion: 4 consecutive counts without a new historical best; improvement resets the counter. The best layout is retained. Fold-specific termination is reported in Table S2. |
| Search DFR | 2 layers, 1 forest per layer, 25 trees, maximum depth 50. |
| Tuned DFR | (layers, forests/layer, trees, max depth): (2,1,25,50), (3,1,50,50), (3,2,50,50). Final output averages the final-layer forests. |
| LR | Ordinary least squares; one candidate, fitted on frame-wise engineered inputs. |
| DNN | Two fully connected ReLU hidden layers: 64/32 or 128/64; linear output. Batch size 4096 frames. |
| LSTM | One unidirectional layer; hidden size 32 or 64; linear output at each frame. Batch size 32 trials. |
| CNN | Two causal Conv1D/ReLU layers: 16 channels with kernel 3, or 32 channels with kernel 5; left padding; kernel-1 linear output. Batch size 32 trials. |
| Neural optimization | Adam; learning rate 0.001; weight decay 0.0001; MSE; maximum 60 epochs; inner early-stopping patience 8. Final epochs = rounded median inner best epoch. |
| Sampling / resources | Every second frame in inner search/tuning scoring; final fitting and testing use all 101 points. Main ARC fold jobs used 12 CPU workers. |

## **Supplementary Table S2. Outer test participants and selected sensor layouts**

Sensors are identified by one-based channel numbers, consistent with the main figures. Each row represents the layout used by one independently trained outer-fold model.

| **Fold** | **Test participants** | **k** | **Selected sensor numbers** |
| --- | --- | --- | --- |
| 1 | S01; S11; S21; S31 | 9 | 2; 5; 43; 78; 94; 102; 184; 223; 230 |
| 2 | S10; S20; S30 | 10 | 9; 28; 56; 62; 96; 109; 124; 126; 149; 183 |
| 3 | S09; S19; S29 | 9 | 6; 8; 11; 84; 88; 101; 107; 170; 171 |
| 4 | S08; S18; S28 | 11 | 9; 44; 59; 98; 101; 118; 143; 147; 185; 186; 209 |
| 5 | S07; S17; S27 | 7 | 10; 11; 41; 84; 92; 138; 197 |
| 6 | S06; S16; S26 | 18 | 6; 16; 26; 27; 28; 58; 74; 98; 102; 107; 165; 172; 174; 176; 181; 221; 223; 231 |
| 7 | S05; S15; S25 | 18 | 6; 7; 8; 16; 40; 48; 56; 80; 82; 84; 95; 120; 128; 132; 149; 178; 185; 222 |
| 8 | S04; S14; S24 | 9 | 10; 24; 48; 61; 78; 84; 135; 175; 195 |
| 9 | S03; S13; S23 | 11 | 9; 44; 59; 84; 88; 132; 163; 171; 186; 188; 212 |
| 10 | S02; S12; S22 | 11 | 1; 3; 32; 37; 46; 84; 124; 134; 197; 218; 223 |

## **Fold-level held-out performance and search termination**

| **Fold** | **Last k** | **Counter** | **R²** | **PCC** | **MAE** | **RMSE** |
| --- | --- | --- | --- | --- | --- | --- |
| 1 | 13 | 4 | 0.790 | 0.891 | 0.037 | 0.052 |
| 2 | 14 | 4 | 0.777 | 0.881 | 0.038 | 0.052 |
| 3 | 13 | 4 | 0.821 | 0.907 | 0.034 | 0.046 |
| 4 | 15 | 4 | 0.780 | 0.889 | 0.038 | 0.056 |
| 5 | 11 | 4 | 0.771 | 0.881 | 0.038 | 0.053 |
| 6 | 20 | 2 | 0.787 | 0.888 | 0.037 | 0.056 |
| 7 | 20 | 2 | 0.878 | 0.938 | 0.028 | 0.038 |
| 8 | 13 | 4 | 0.732 | 0.857 | 0.039 | 0.055 |
| 9 | 15 | 4 | 0.779 | 0.885 | 0.037 | 0.053 |
| 10 | 15 | 4 | 0.831 | 0.913 | 0.034 | 0.048 |

Counter is the number of consecutive counts without a new historical best at termination. Folds 6 and 7 reached the count cap with counter 2; the other folds met patience 4. All 10 final fold predictions are included.

## **Supplementary Table S3. Pooled held-out model performance**

Each metric combines all held-out frames for the indicated movement, or all four movements for the overall row. Main Table 1 separately reports fold means. Each participant contributes test predictions from only one outer fold. MAE and RMSE are in N·m/N.

| **Movement** | **Model** | **R²** | **PCC** | **MAE** | **RMSE** |
| --- | --- | --- | --- | --- | --- |
| Overall movements | DFR | 0.7946 | 0.8920 | 0.0362 | 0.0512 |
|  | LR | 0.5986 | 0.7738 | 0.0542 | 0.0716 |
|  | LSTM | 0.8009 | 0.8955 | 0.0369 | 0.0504 |
|  | DNN | 0.7966 | 0.8946 | 0.0370 | 0.0510 |
|  | CNN | 0.7305 | 0.8552 | 0.0435 | 0.0587 |
| 90° cutting | DFR | 0.8632 | 0.9298 | 0.0358 | 0.0485 |
|  | LR | 0.6861 | 0.8287 | 0.0567 | 0.0735 |
|  | LSTM | 0.8608 | 0.9281 | 0.0368 | 0.0489 |
|  | DNN | 0.8649 | 0.9319 | 0.0361 | 0.0482 |
|  | CNN | 0.8050 | 0.8981 | 0.0433 | 0.0579 |
| Sprinting | DFR | 0.7587 | 0.8713 | 0.0372 | 0.0540 |
|  | LR | 0.5469 | 0.7397 | 0.0562 | 0.0739 |
|  | LSTM | 0.7937 | 0.8913 | 0.0362 | 0.0499 |
|  | DNN | 0.7756 | 0.8816 | 0.0376 | 0.0520 |
|  | CNN | 0.6995 | 0.8370 | 0.0439 | 0.0602 |
| Running pass | DFR | 0.7667 | 0.8766 | 0.0329 | 0.0463 |
|  | LR | 0.5290 | 0.7281 | 0.0489 | 0.0658 |
|  | LSTM | 0.7665 | 0.8773 | 0.0339 | 0.0463 |
|  | DNN | 0.7641 | 0.8771 | 0.0343 | 0.0466 |
|  | CNN | 0.6949 | 0.8344 | 0.0401 | 0.0529 |
| 180° COD | DFR | 0.7172 | 0.8474 | 0.0390 | 0.0555 |
|  | LR | 0.5116 | 0.7210 | 0.0550 | 0.0729 |
|  | LSTM | 0.7122 | 0.8453 | 0.0407 | 0.0560 |
|  | DNN | 0.7074 | 0.8454 | 0.0400 | 0.0564 |
|  | CNN | 0.6345 | 0.7996 | 0.0468 | 0.0631 |

## **Supplementary Table S4. Paired participant-level omnibus model comparisons**

N = 31 participants, five models, and four degrees of freedom for each Friedman test. Holm adjustment is across the three omnibus metrics; Kendall’s W quantifies the rank effect.

| **Metric** | **χ²** | **df** | **Kendall W** | **Raw p** | **Holm p** |
| --- | --- | --- | --- | --- | --- |
| R² | 88.748 | 4 | 0.716 | 2.43e-18 | 4.86e-18 |
| RMSE | 88.748 | 4 | 0.716 | 2.43e-18 | 4.86e-18 |
| MAE | 95.252 | 4 | 0.768 | 1.01e-19 | 3.02e-19 |

## **Supplementary Table S5. Paired comparisons of DFR with alternative models**

Two-sided Wilcoxon signed-rank tests; N = 31. Positive median advantage and rank-biserial correlation favor DFR (higher R² or lower error). Holm adjustment is across the 12 DFR-model/metric comparisons.

| **Metric** | **Other** | **W** | **Median advantage** | **Rank-biserial r** | **Raw p** | **Holm p** |
| --- | --- | --- | --- | --- | --- | --- |
| R² | LR | 0.0 | 0.2009 | 1.000 | 9.31e-10 | 1.12e-08 |
| R² | DNN | 211.0 | -0.0113 | -0.149 | 0.480 | 1.000 |
| R² | LSTM | 207.0 | -0.0095 | -0.165 | 0.433 | 1.000 |
| R² | CNN | 1.0 | 0.0644 | 0.996 | 1.86e-09 | 1.49e-08 |
| RMSE | LR | 0.0 | 0.0196 | 1.000 | 9.31e-10 | 1.12e-08 |
| RMSE | DNN | 213.0 | -0.0013 | -0.141 | 0.504 | 1.000 |
| RMSE | LSTM | 200.0 | -0.0017 | -0.194 | 0.357 | 1.000 |
| RMSE | CNN | 1.0 | 0.0066 | 0.996 | 1.86e-09 | 1.49e-08 |
| MAE | LR | 0.0 | 0.0181 | 1.000 | 9.31e-10 | 1.12e-08 |
| MAE | DNN | 178.0 | 0.0005 | 0.282 | 0.176 | 1.000 |
| MAE | LSTM | 203.0 | 0.0002 | 0.181 | 0.388 | 1.000 |
| MAE | CNN | 0.0 | 0.0070 | 1.000 | 9.31e-10 | 1.12e-08 |

## **Supplementary Table S6. Participant and trial distributions of DFR prediction error**

Values are median [Q1, Q3]. Participant summaries use all held-out frames for that participant and movement. Trial summaries use one 101-point waveform. RMSE and MAE are in N·m/N. Ranges give the minimum and maximum values.

## **Participants**

| **Movement** | **Metric** | **N** | **Median [IQR]** | **Range** |
| --- | --- | --- | --- | --- |
| Overall movements | R² | 31 | 0.800 [0.735, 0.838] | 0.664 to 0.905 |
|  | PCC | 31 | 0.895 [0.866, 0.919] | 0.836 to 0.952 |
|  | MAE | 31 | 0.036 [0.035, 0.038] | 0.026 to 0.043 |
|  | RMSE | 31 | 0.051 [0.048, 0.056] | 0.035 to 0.068 |
| 90° cutting | R² | 30 | 0.852 [0.824, 0.896] | 0.661 to 0.936 |
|  | PCC | 30 | 0.937 [0.917, 0.950] | 0.851 to 0.971 |
|  | MAE | 30 | 0.036 [0.031, 0.040] | 0.024 to 0.049 |
|  | RMSE | 30 | 0.048 [0.041, 0.054] | 0.030 to 0.067 |
| Sprinting | R² | 31 | 0.789 [0.710, 0.833] | -0.539 to 0.940 |
|  | PCC | 31 | 0.905 [0.873, 0.924] | 0.710 to 0.978 |
|  | MAE | 31 | 0.034 [0.029, 0.046] | 0.018 to 0.065 |
|  | RMSE | 31 | 0.046 [0.040, 0.061] | 0.023 to 0.097 |
| Running pass | R² | 31 | 0.753 [0.663, 0.804] | 0.248 to 0.943 |
|  | PCC | 31 | 0.882 [0.849, 0.912] | 0.631 to 0.975 |
|  | MAE | 31 | 0.032 [0.027, 0.037] | 0.021 to 0.049 |
|  | RMSE | 31 | 0.044 [0.036, 0.051] | 0.028 to 0.067 |
| 180° COD | R² | 30 | 0.728 [0.632, 0.803] | 0.271 to 0.920 |
|  | PCC | 30 | 0.860 [0.799, 0.907] | 0.588 to 0.961 |
|  | MAE | 30 | 0.040 [0.031, 0.042] | 0.021 to 0.061 |
|  | RMSE | 30 | 0.051 [0.042, 0.060] | 0.028 to 0.103 |

## **Trials**

| **Movement** | **Metric** | **N** | **Median [IQR]** | **Range** |
| --- | --- | --- | --- | --- |
| Overall movements | R² | 590 | 0.784 [0.613, 0.882] | -64.542 to 0.982 |
|  | PCC | 590 | 0.933 [0.888, 0.963] | -0.751 to 0.995 |
|  | MAE | 590 | 0.031 [0.023, 0.045] | 0.008 to 0.197 |
|  | RMSE | 590 | 0.040 [0.029, 0.055] | 0.010 to 0.224 |
| 90° cutting | R² | 147 | 0.853 [0.760, 0.913] | -0.818 to 0.977 |
|  | PCC | 147 | 0.955 [0.922, 0.976] | 0.298 to 0.995 |
|  | MAE | 147 | 0.034 [0.026, 0.042] | 0.012 to 0.113 |
|  | RMSE | 147 | 0.044 [0.033, 0.052] | 0.015 to 0.135 |
| Sprinting | R² | 145 | 0.804 [0.665, 0.882] | -17.346 to 0.979 |
|  | PCC | 145 | 0.938 [0.895, 0.962] | -0.751 to 0.994 |
|  | MAE | 145 | 0.031 [0.022, 0.045] | 0.012 to 0.132 |
|  | RMSE | 145 | 0.039 [0.029, 0.057] | 0.015 to 0.174 |
| Running pass | R² | 148 | 0.745 [0.540, 0.833] | -22.652 to 0.972 |
|  | PCC | 148 | 0.921 [0.854, 0.958] | -0.361 to 0.992 |
|  | MAE | 148 | 0.028 [0.021, 0.042] | 0.009 to 0.088 |
|  | RMSE | 148 | 0.036 [0.028, 0.054] | 0.011 to 0.105 |
| 180° COD | R² | 150 | 0.692 [0.398, 0.845] | -64.542 to 0.982 |
|  | PCC | 150 | 0.917 [0.849, 0.954] | -0.541 to 0.991 |
|  | MAE | 150 | 0.033 [0.023, 0.049] | 0.008 to 0.197 |
|  | RMSE | 150 | 0.040 [0.029, 0.063] | 0.010 to 0.224 |

Trial R² can be strongly negative when errors are large relative to measured within-trial variation. The overall trial mean was 0.430 and the median was 0.784; medians, IQRs, and ranges summarize this skewed distribution.

## **Supplementary Table S7. Waveform normalization and peak agreement**

| **Movement** | **Metric** | **N** | **Median [Q1, Q3]** |
| --- | --- | --- | --- |
| Overall movements | NRMSE (%) | 590 | 15.1913 [11.6449, 19.9071] |
|  | Absolute peak error | 590 | 0.0329 [0.0145, 0.0565] |
|  | Absolute timing error (percentage points) | 590 | 6.0000 [2.0000, 13.7500] |
| 90° cutting | NRMSE (%) | 147 | 13.0823 [9.9135, 16.1277] |
|  | Absolute peak error | 147 | 0.0331 [0.0132, 0.0568] |
|  | Absolute timing error (percentage points) | 147 | 5.0000 [2.0000, 12.0000] |
| Sprinting | NRMSE (%) | 145 | 14.6062 [11.9471, 19.7243] |
|  | Absolute peak error | 145 | 0.0305 [0.0132, 0.0530] |
|  | Absolute timing error (percentage points) | 145 | 7.0000 [3.0000, 15.0000] |
| Running pass | NRMSE (%) | 148 | 17.0396 [13.8777, 22.5747] |
|  | Absolute peak error | 148 | 0.0352 [0.0166, 0.0543] |
|  | Absolute timing error (percentage points) | 148 | 8.5000 [3.0000, 14.0000] |
| 180° COD | NRMSE (%) | 150 | 15.8924 [12.2347, 22.3549] |
|  | Absolute peak error | 150 | 0.0319 [0.0167, 0.0587] |
|  | Absolute timing error (percentage points) | 150 | 3.0000 [1.0000, 11.0000] |

## **Participant-level peak agreement by movement**

| **Movement** | **N** | **Bias** | **95% limits of agreement** | **CCC** |
| --- | --- | --- | --- | --- |
| 90° cutting | 30 | -0.0145 | -0.0570, 0.0280 | 0.814 |
| Sprinting | 31 | -0.0024 | -0.0630, 0.0582 | 0.669 |
| Running pass | 31 | -0.0024 | -0.0539, 0.0492 | 0.702 |
| 180° COD | 30 | -0.0015 | -0.0384, 0.0354 | 0.944 |

Peak = maximum over the trial waveform. Bias = predicted minus measured mean trial peak within each participant and movement. Limits of agreement = bias ± 1.96 × sample SD of the participant differences. CCC is the concordance correlation coefficient. Peak errors and agreement are in N·m/N. Absolute timing error is in percentage points of stance. Participant counts reflect available accepted trials by movement.

## **Supplementary Table S8. Fold-level prediction accuracy for the three knee-moment components**

The components represent sagittal-plane flexion-extension, frontal-plane adduction-abduction, and transverse-plane internal-external rotation. The component and resultant analyses used the same held-out participants. Each component model was tuned separately within the outer-training participants using the sensor layout selected for the resultant in that fold. MAE and RMSE are in N·m/N. Values are means (sample SD) across the 10 outer test folds. Pooled component values are reported separately in main Section 3.4 and Table 2.

| **Component** | **Summary** | **R²** | **PCC** | **MAE** | **RMSE** |
| --- | --- | --- | --- | --- | --- |
| Flexion-extension | Mean (sample SD) | 0.8057 (0.0474) | 0.8990 (0.0258) | 0.0404 (0.0040) | 0.0571 (0.0069) |
| Adduction-abduction | Mean (sample SD) | 0.6406 (0.0948) | 0.8051 (0.0548) | 0.0279 (0.0031) | 0.0396 (0.0053) |
| Internal-external rotation | Mean (sample SD) | 0.6341 (0.1453) | 0.7960 (0.0916) | 0.0160 (0.0020) | 0.0256 (0.0042) |

**Supplementary Table S9. Amplitude and timing errors of knee-moment component extrema**

| **Movement** | **Component** | **Extremum** | **Amplitude error (N·m/N)** | **Timing error (stance pp)** | **Measured at endpoint (%)** |
| --- | --- | --- | --- | --- | --- |
| All movements (n = 590) | Flexion-extension | Maximum | 0.0311 [0.0142, 0.0549] | 6.0 [2.0, 14.8] | 0.7 |
|  | Flexion-extension | Minimum | 0.0218 [0.0092, 0.0390] | 1.0 [0.0, 3.0] | 79.2 |
|  | Adduction-abduction | Maximum | 0.0195 [0.0084, 0.0373] | 7.5 [2.0, 28.0] | 14.6 |
|  | Adduction-abduction | Minimum | 0.0190 [0.0087, 0.0358] | 3.0 [1.0, 16.0] | 25.9 |
|  | Internal-external rotation | Maximum | 0.0092 [0.0040, 0.0195] | 3.0 [1.0, 13.0] | 28.3 |
|  | Internal-external rotation | Minimum | 0.0134 [0.0053, 0.0239] | 11.0 [4.0, 21.0] | 2.2 |
| 90° cutting (n = 147) | Flexion-extension | Maximum | 0.0358 [0.0167, 0.0560] | 6.0 [2.0, 12.0] | 0.7 |
|  | Flexion-extension | Minimum | 0.0256 [0.0137, 0.0411] | 1.0 [0.0, 2.0] | 79.6 |
|  | Adduction-abduction | Maximum | 0.0208 [0.0078, 0.0379] | 7.0 [1.5, 37.5] | 23.1 |
|  | Adduction-abduction | Minimum | 0.0229 [0.0091, 0.0438] | 2.0 [1.0, 12.0] | 7.5 |
|  | Internal-external rotation | Maximum | 0.0129 [0.0052, 0.0246] | 3.0 [1.0, 8.0] | 12.2 |
|  | Internal-external rotation | Minimum | 0.0152 [0.0061, 0.0268] | 12.0 [4.0, 23.0] | 3.4 |
| Sprinting (n = 145) | Flexion-extension | Maximum | 0.0245 [0.0135, 0.0458] | 8.0 [3.0, 16.0] | 0.7 |
|  | Flexion-extension | Minimum | 0.0261 [0.0104, 0.0456] | 1.0 [0.0, 5.0] | 68.3 |
|  | Adduction-abduction | Maximum | 0.0184 [0.0082, 0.0365] | 6.0 [3.0, 18.0] | 2.8 |
|  | Adduction-abduction | Minimum | 0.0151 [0.0072, 0.0295] | 4.0 [1.0, 28.0] | 57.9 |
|  | Internal-external rotation | Maximum | 0.0049 [0.0020, 0.0087] | 6.0 [2.0, 48.0] | 36.6 |
|  | Internal-external rotation | Minimum | 0.0114 [0.0052, 0.0197] | 6.0 [2.0, 15.0] | 4.1 |

Values are median [Q1, Q3] across all retained trials; n denotes trials. Cutting and 180° COD include 30 participants; sprinting and running pass include 31. Maximum and minimum are calculated independently for measured and predicted signed waveforms over 101 points, using the first occurrence for exact ties. Errors are absolute differences in amplitude or location; timing is in percentage points of stance. Endpoint denotes 0% or 100%.

**Supplementary Table S9. Amplitude and timing errors of knee-moment component extrema (continued)**

| **Movement** | **Component** | **Extremum** | **Amplitude error (N·m/N)** | **Timing error (stance pp)** | **Measured at endpoint (%)** |
| --- | --- | --- | --- | --- | --- |
| Running pass (n = 148) | Flexion-extension | Maximum | 0.0361 [0.0157, 0.0599] | 7.5 [3.0, 14.0] | 0.0 |
|  | Flexion-extension | Minimum | 0.0235 [0.0097, 0.0401] | 1.0 [0.0, 2.0] | 85.8 |
|  | Adduction-abduction | Maximum | 0.0154 [0.0081, 0.0307] | 7.0 [3.0, 20.0] | 6.8 |
|  | Adduction-abduction | Minimum | 0.0128 [0.0068, 0.0234] | 3.0 [1.0, 7.0] | 29.7 |
|  | Internal-external rotation | Maximum | 0.0098 [0.0048, 0.0168] | 3.0 [1.0, 7.2] | 40.5 |
|  | Internal-external rotation | Minimum | 0.0136 [0.0049, 0.0242] | 10.0 [4.0, 20.0] | 0.7 |
| 180° COD (n = 150) | Flexion-extension | Maximum | 0.0291 [0.0120, 0.0553] | 4.0 [1.2, 16.8] | 1.3 |
|  | Flexion-extension | Minimum | 0.0151 [0.0053, 0.0285] | 1.0 [0.0, 4.0] | 82.7 |
|  | Adduction-abduction | Maximum | 0.0228 [0.0105, 0.0416] | 12.0 [1.0, 43.5] | 25.3 |
|  | Adduction-abduction | Minimum | 0.0283 [0.0149, 0.0498] | 4.0 [1.0, 40.0] | 9.3 |
|  | Internal-external rotation | Maximum | 0.0139 [0.0054, 0.0228] | 2.0 [1.0, 15.0] | 24.0 |
|  | Internal-external rotation | Minimum | 0.0160 [0.0049, 0.0293] | 14.5 [6.0, 29.5] | 0.7 |

Values are median [Q1, Q3] across all retained trials; n denotes trials. Cutting and 180° COD include 30 participants; sprinting and running pass include 31. Maximum and minimum are calculated independently for measured and predicted signed waveforms over 101 points, using the first occurrence for exact ties. Errors are absolute differences in amplitude or location; timing is in percentage points of stance. Endpoint denotes 0% or 100%.

**Supplementary Table S10. Stance intervals with above-mean sensor contribution to resultant predictions**

| **Summary** | **Trials** | **Curve mean (N·m/N)** | **Above-mean stance points (%)** | **Maximum location (%)** |
| --- | --- | --- | --- | --- |
| All movements | 590 | 0.138863 | 9-55 | 24 |
| 90° cutting | 147 | 0.169799 | 10-41, 78-98 | 13 |
| Sprinting | 145 | 0.139135 | 7-57, 59 | 11 |
| Running pass | 148 | 0.115897 | 9-60 | 26 |
| 180° COD | 150 | 0.130941 | 12-36, 41-54, 57-58, 87-96 | 21 |

Absolute SHAP was summed over sensor groups and averaged across trials at each target frame. The cutoff is the arithmetic mean of the resulting 101-point curve, calculated separately for the pooled data and each movement. Consecutive points strictly above the cutoff define each reported interval; isolated points and gaps are retained. These intervals summarize model-attribution magnitude.

## **Supplementary Figure S1. Laboratory arrangement and marker configuration**

Upper panel: overview of the acquisition area, with the 90 × 60 cm infill turf-covered platform indicated. Lower-left panel: front view of the participant showing marker placement. Lower-right panel: the infill turf-covered section (488 × 183 cm), screw fixation of the platform to the force plate, and an approximately 1 cm gap from adjacent platforms. Surrounding areas were covered with non-infill turf to simulate a rugby training and match environment. The force plate was zeroed after installation of the platform and turf. Four-marker clusters were used on each thigh, shank, and rearfoot; each forefoot cluster contained three markers. Artificial-turf construction and sampling frequencies are detailed in Section 2.2.

## **Supplementary Figure S2. Trial-level waveform and peak-error distributions for the four rugby-specific movements.**

Boxes show the median and interquartile range, whiskers extend to 1.5 times the interquartile range, and points show individual outer-test trials. NRMSE was 100 × trial waveform RMSE divided by the observed within-trial peak-to-peak range. Peak magnitude is the maximum resultant moment within the 101-point stance-normalized waveform, and peak timing error is expressed in percentage points of stance. The distributions summarize trials nested within participants.

## **Supplementary Figure S3. Participant-level Bland-Altman agreement for peak knee resultant moment by movement.**

Each point represents one participant's mean trial peak within the indicated movement (n = 30 or 31). The solid line denotes the mean difference (predicted minus observed), and dashed lines denote the 95% limits of agreement calculated as the mean difference ± 1.96 times the sample standard deviation of the differences.

## **Supplementary Figure S4. Between-participant variability in measured and predicted knee resultant-moment waveforms.**

Repeated trials were first averaged within each participant and movement. Lines show the between-participant sample standard deviation at each of the 101 stance-normalized points. The inset values report the ratio of the mean predicted SD to the mean measured SD and the number of stance points at which predicted SD was lower than measured SD.

Supplementary Figure S5. Participant-level errors in signed knee-moment component extrema. Columns show flexion-extension, adduction-abduction, and internal-external rotation moments. (A-C) Absolute extremum-amplitude error (N·m/N). (D-F) Absolute extremum-timing error (percentage points of stance). Maxima and minima are determined independently from each measured and predicted signed trial waveform over the 101 stance points; exact ties use the first occurrence. Each point summarizes one participant's median trial error across all available movements (31 participants, 590 trials overall). Boxes show medians and interquartile ranges across participant medians; whiskers show their full range. Global extrema include stance endpoints. Trial-level results overall and by movement, including endpoint fractions, are reported in Supplementary Table S9.

Supplementary Figure S6. Pooled temporal and spatial contributions to held-out resultant-moment predictions. (A) Total absolute sensor contributions at each target stance point, averaged across 590 trials and all four movements. The red dashed interval marks points above the 101-point curve's arithmetic mean, with a maximum at 24% of stance. (B) Spatial map of the 80-sensor union, with one-based sensor numbers and conditional mean absolute SHAP colors. Spatial means use only the folds in which each sensor was selected, with equal weight across those folds. Contributions were estimated from the full fitted DFR cascade for the resultant, conditional on movement. Full layouts are provided in Supplementary Table S2.
